# Hyperparathyroidism jaw tumour syndrome: a pictoral review

**DOI:** 10.1007/s13244-016-0519-0

**Published:** 2016-09-21

**Authors:** Hannah du Preez, Ashok Adams, Polly Richards, Simon Whitley

**Affiliations:** 1 Department of Radiology, Barts and The Royal London Hospitals, Barts Health NHS, London, UK; 2 Department of Oral and Maxillofacial surgery, Barts and The Royal London Hospitals, Barts Health NHS, London, UK

**Keywords:** Hyperparathyroidism, Jaw neoplasms, Primary, Parathyroid neoplasms, Fibroma, Ossifying

## Abstract

**Abstract:**

Hyperparathyroidism jaw tumour syndrome is a rare autosomal dominant inherited endocrine neoplasia syndrome, which predisposes carriers to develop a triad of multiple ossifying fibromas of the maxilla and mandible, parathyroid adenomas and carcinomas (with consequent hyperparathyroidism) as well as renal and uterine tumours. The prevalence of this condition is unknown. Patients typically present initially with symptoms and signs of a jaw tumour. A high index of suspicion is required for the underlying diagnosis to be recognised, enabling appropriate management of jaw lesions, treatment of hyperparathyroidism, if present, as well as early detection of malignant disease and screening of family members.

***Teaching points*:**

• *HPT-JT is a rare autosomal dominant inherited endocrine neoplasia syndrome*.

• *HPT-JT causes facial disfigurement, morbidity secondary to hyperparathyroidism and malignancy*.

• *Patients can present with ossifying fibromas of the jaw, hypercalcaemia or malignancy*.

• *A high index of suspicion is required for the underlying diagnosis to be recognised*.

• *Management involves screening of family members*.

## Introduction

Hyperparathyroidism jaw tumour syndrome (HPT-JT) is a rare autosomal dominant inherited endocrine neoplasia syndrome, which predisposes carriers to develop a triad of multiple ossifying fibromas of the maxilla and mandible, parathyroid adenomas and carcinomas (with consequent hyperparathyroidism), as well as renal and uterine tumours.

### Genetics

HPT-JT is inherited in an autosomal dominant pattern and can demonstrate incomplete penetrance. Instances of sporadic mutations arising de novo have been recorded [1]. The syndrome arises from a gene mutation, which inhibits the production of normal parafibromin, a protein that regulates cell proliferation and can arrest the cell cycle [1]. The gene responsible for parafibromin expression is the HRPT2 tumour suppressor gene (also known as the CDC73 gene), which has been localised to a 34-cM region on 1q25 - q31 [2]. Mutations of HRPT2 are identified in most individuals affected by HPT-JT (and many of their asymptomatic family members) [3]. Loss of normal parafibromin expression allows certain tissues to propagate unimpeded. Gene sequencing is of use in confirming the diagnosis and differentiating the disease from other inherited endocrine neoplasia syndromes which also have parathyroid manifestations (e.g., multiple endocrine neoplasia (MEN) types I and II).

### Clinical presentation

Individuals with HPT-JT typically present with jaw and teeth deformities secondary to ossifying fibromas of the maxilla and mandible. These produce a hard, deforming jaw swelling, which can cause early tooth displacement. Alternatively, they may present with symptoms and signs of hypercalcaemia secondary to hyperparathyroidism due to an underlying parathyroid adenoma or carcinoma. A few patients will also be detected incidentally with a high serum calcium on blood biochemistry that subsequently prompts further investigation. Unfortunately, some patients can also present with the sequelae of renal, uterine or parathyroid malignancy.

### Imaging findings

**Fig. 1:**
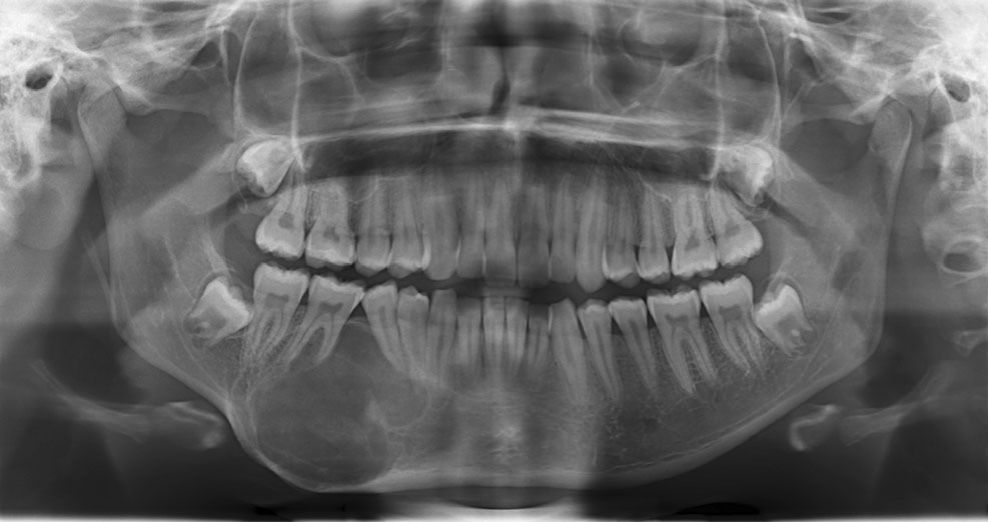
An orthopantomogram of an 18-year-old female that demonstrates an ossifying fibroma of the right body of the mandible. The solitary expansile lesion splays the teeth and has a discernible sclerotic rim, but no destructive features, e.g., root resorption

**Fig. 2:**
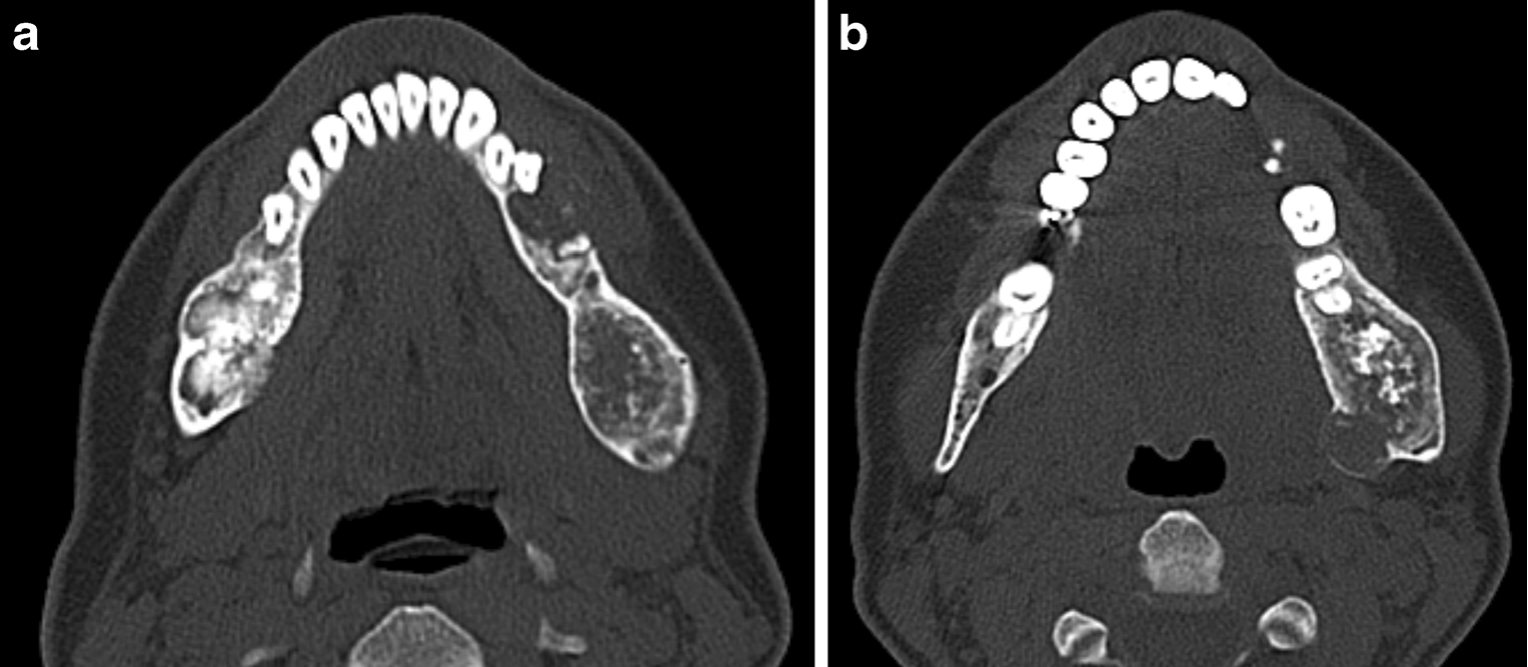
**a**), **b**) Axial 1.5 mm CT images through the mandible of a 30-year-old female with HPT-JT. There are multiple bilateral ossifying fibromas of the mandible, as is typical of the condition. Ossifying fibromas are composed of fibrous tissue with varying amounts of mature bone, and; therefore, as demonstrated here, some lesions are purely lucent, while others will have a more conspicuous calcified component
  Ossifying fibromas of the jaw are benign lesions with the risk of malignant degeneration being less than <0.5 % [5]. However, they demonstrate locally invasive features, e.g., they may splay the teeth [4]. They are expansile and can cause facial asymmetry. They can extend into the maxillary sinus (Fig. 3). Extension into the orbital floor may precipitate periorbital swelling, orbital pain or changes in visual acuity (Fig. 4). If left untreated, ossifying fibromas may continue to enlarge and become severely disfiguring (Fig. 5).

i. Ossifying fibromas
  Ossifying fibromas of the jaw are a type of benign fibro-osseous lesion detected in approximately 50 % of HPT-JT individuals and present in adolescents or young adults [2]. In contrast, sporadic ossifying fibromas of the jaw are rare and typically occur in an older age group [4]. Ossifying fibromas of the jaw arise from the mesenchymal cells of the periodontal ligament, the fibrous connective tissue found within the periodontal space, which secures the tooth [5]. They are composed of fibrous tissue with varying amounts of mature bone, osteoid and cementum [6] .
  On plain film and CT ossifying fibromas appear as well-circumscribed expansile lesions arising within the mandible or maxilla (Fig. 1). They are commonly uniocular, but can be multilocular and demonstrate sizeable growth (greater than 10 cm) [5]. On plain film imaging, they are usually lucent, but can demonstrate a mixed opacity or be completely radio-opaque, depending on the relative fibrous and calcified components [7]. They typically have a sclerotic rim. On CT they demonstrate internal soft tissue density, which reflects the fibrous component (Fig. 2). On MR T1-weighted imaging they return low to intermediate signal, the low signal areas reflecting the osseous component. On T2-weighted imaging, they contain a mixture of low and high signal components reflecting the osseous and fibrous components, respectively [6]. The fibrous component of the tumour enhances following administration of contrast [6]. Ossifying fibromas typically do not cause root absorption, which is a feature of more aggressive lesions such as ameloblastomas, and unlike radicular or dentigerous cysts, there is no association with unerupted teeth [4].

**Fig. 3:**
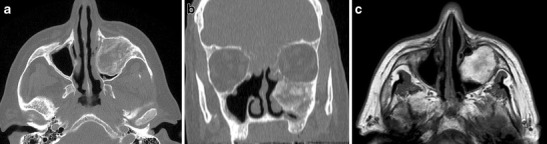
**a**) Axial and **b**) coronal CT images with **c**) TI-weighted axial MRI at the level of the maxillary sinuses in a 60-yr-old male with HPT-JT. The patient presented with symptoms of chronic sinusitis and CT imaging demonstrates an ossifying fibroma which arises from the maxilla and almost obliterates the left maxillary sinus. On MR the imaging features are those of a partially ossified expansile fatty soft tissue mass, areas of focal low signal on the T1 weighted sequence reflect the calcified osseous components of the ossifying fibroma. There is evidence of previous sinonasal surgery with wide bilateral medial antrostomies, middle turbinectomies, uncinectomies and ethmoidectomies. Opacification, suggestive of a mucocele, was noted in the anterior ethmoid and frontal sinuses

**Fig. 4:**
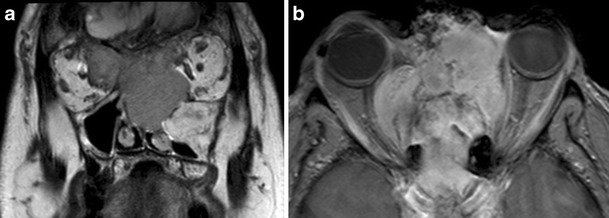
**a**) Coronal T2-weighted and **b**) axial post-contrast T1 fat-saturated MR images. The patient from Fig. 3 subsequently represented two years later with difficulty opening his right eye and changes in visual acuity. MRI demonstrated an enhancing mass lesion centered on the ethmoid sinuses, naso ethmoid complex and cribiform plate, which invades both orbits. There is invasion of the intraconal space of the right orbit, displacement of the right optic nerve and inferior lateral displacement of the right globe. The lesion was proven on biopsy to be an ossifying fibroma, although it behaved atypically in an invasive manner

**Fig. 5:**
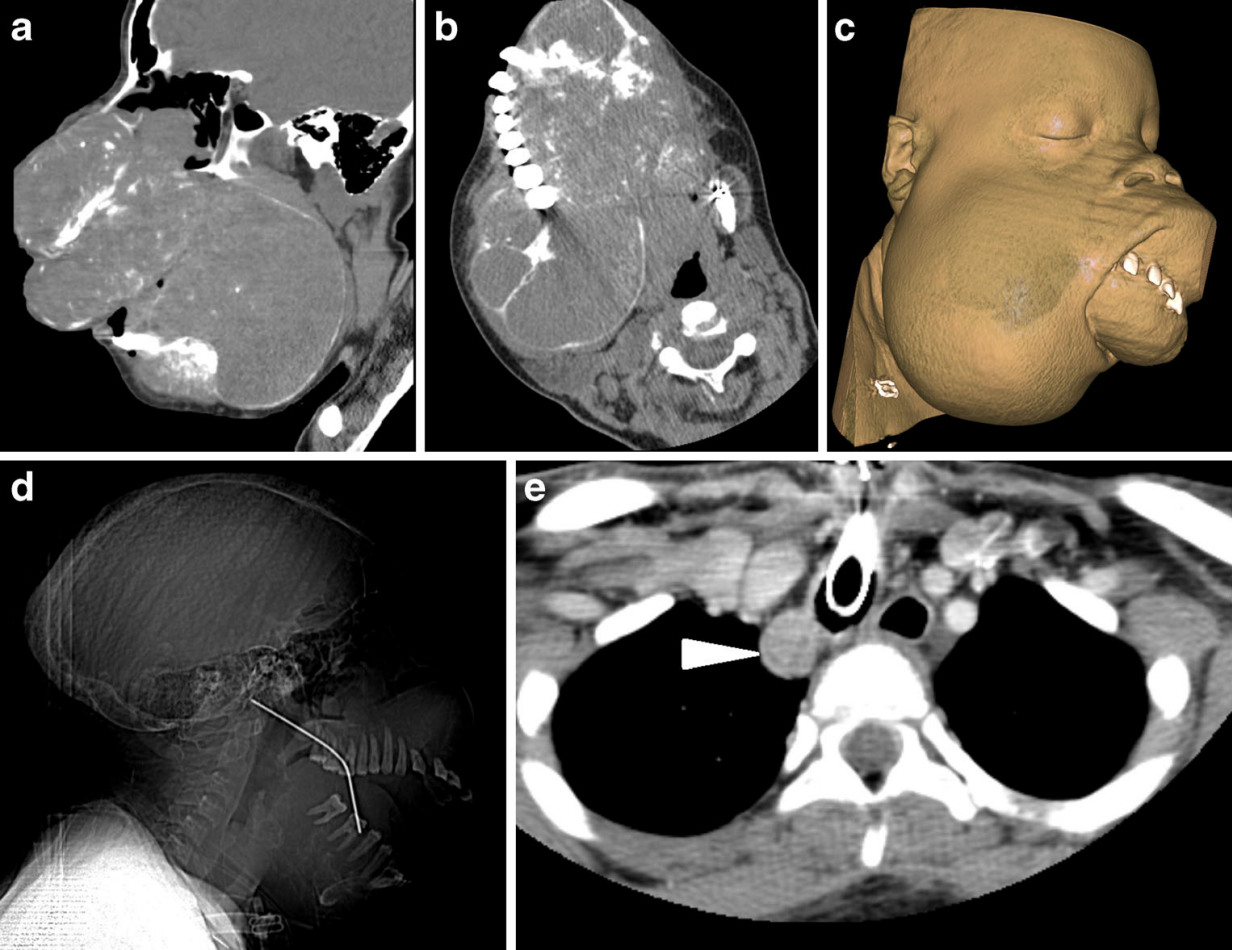
**a**) Midline sagittal CT image **b**) axial CT image at the level of maxilla with **c**) CT 3-D soft tissue volume rendered image and **d**) sagittal CT tomogram of a 19-yr-old female from an east African country. This case illustrates how deforming HPT-JT can become in an environment with poor access to healthcare resources. This patient’s underlying diagnosis was not made prior to her presentation in the UK for debulking surgery. There are extensive ossifying fibromas affecting the entire craniofacial skeleton causing considerable disfigurement of the facial features, encroaching on the orbits and compromising her airway to the extent that she required a tracheostomy. There is evidence of previous surgical intervention in the form of a right jaw strut. **e**) Axial CT through the upper thorax. On her initial CT incidental note was made of an enhancing ovoid lesion in the right paratracheal recess (arrowhead), the underlying diagnosis of HPT-JT was suggested by the radiologist, and this lesion was subsequently proven to be a parathyroid adenoma
  The distinction from fibrous dysplasia and osteolytic Brown tumours of hyperparathyroidism is an important one as both conditions may also arise in the jaw. Fibrous dysplasia produces ill-defined bony expansion and has a characteristic ‘ground glass’ appearance, but it can be indistinguishable from an ossifying fibroma on imaging (Fig. 6) underlining the importance of multidisciplinary team discussion in making the diagnosis of an ossifying fibroma, and the need to review and correlate clinical, imaging and histopathological evidence [4]. As with ossifying fibromas of HPT-JT, Brown tumours may also appear on imaging as lucent jaw lesions with evidence of hyperparathyroid bone disease in the surrounding bone [7]. However, Brown tumours are purely lytic lesions, which lack the sclerotic rim associated with ossifying fibromas. Other important differentials are cementomas, which appear uniformly dense when mature, and osteosarcomas, which appear as ill-defined destructive lesions characterized by aggressive periosteal reaction [6].

**Fig. 6:**
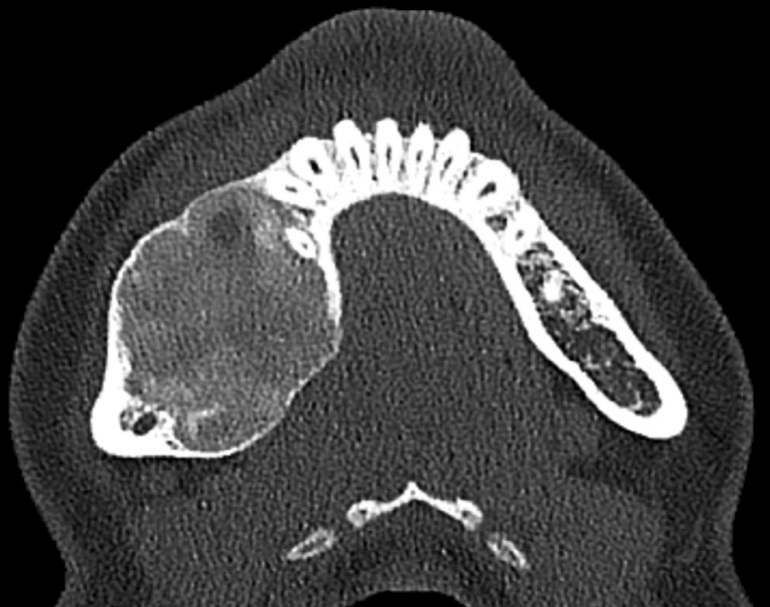
Axial 1.5 mm CT bone reformats of mandible. There is wide variation in the imaging appearances of ossifying fibromas, often the diagnosis of lucent jaw lesions will only be made following MDT discussion and histopathology sampling. This ossifying fibroma of the right body of the mandible demonstrates the internal ground glass texture more commonly associated with fibrous dysplasia, which, along with Browns tumours, are important differentials for a suspected ossifying fibroma
ii. Parathyroid adenomas and carcinomas
  HPT-JT patients with parathyroid adenomas or carcinomas will develop primary hyperparathyroidism and consequent hypercalcaemia. Patients may present with typical but non-specific symptoms of hypercalcaemia including malaise, depression, renal stones and abdominal pain or incidentally identified following identification of a raised serum calcium on blood biochemistry. Hyperparathyroid bone disease is characterised by subperiosteal bone resorption, sclerotic bone changes and/or terminal tuft reabsorption (Fig. 7) [8]. The presence of jaw lesions in combination with bone changes suggestive of hyperparathyroid bone disease should prompt the radiologist to consider HPT-JT and review for a parathyroid lesion (Fig. 8).

**Fig. 7:**
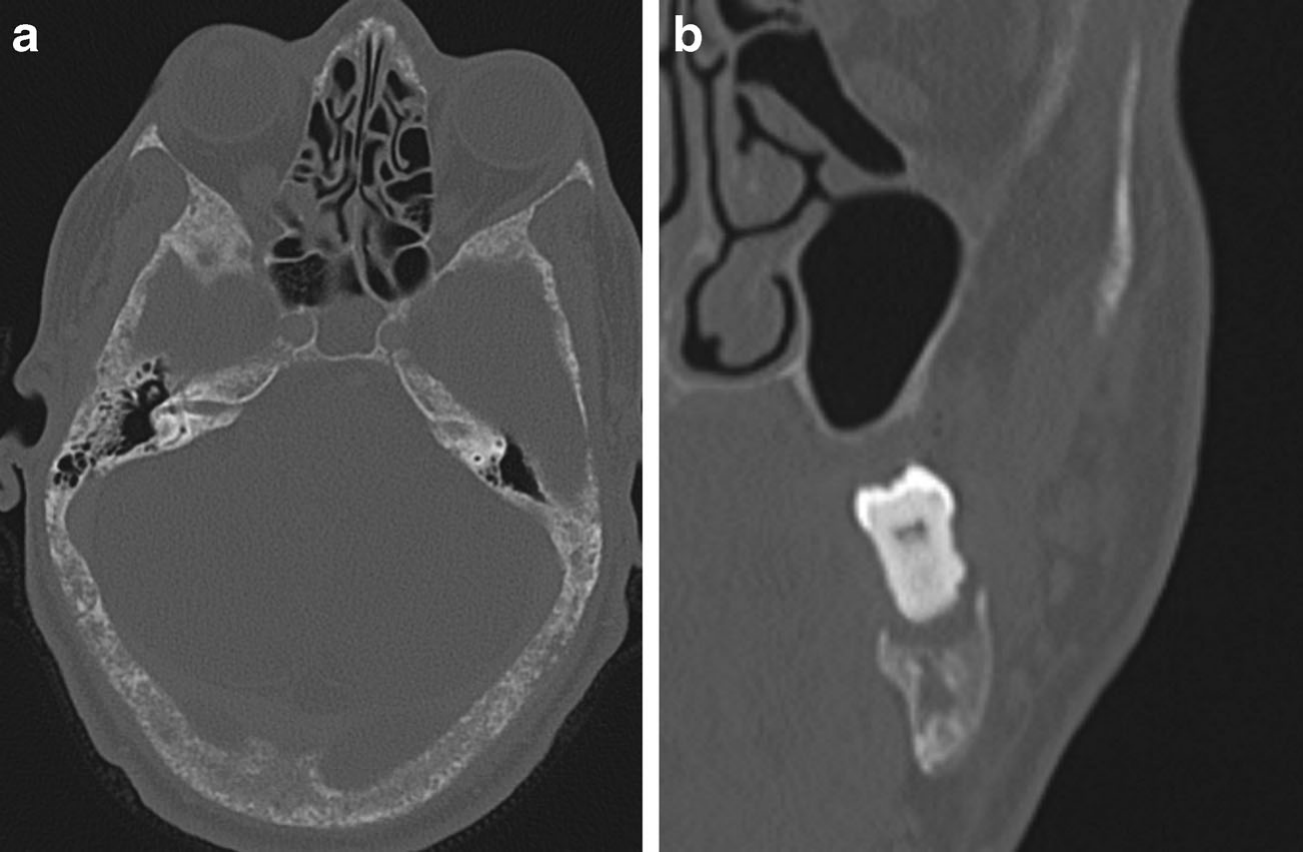
**a**) Axial CT bone reformat through skull base **b**) coronal CT bone reformat of left mandible in a 50-year-old male with HPT-JT and extensive hyperparathyroid bone disease. There is a ‘salt and pepper’ appearance to the skull base secondary to trabecular bone reabsorption. Subperiosteal bone reabsorption of the lamina dura gives a floating appearance to a left lower molar tooth (b). There has been substantial maxillary bone loss and loss of the upper left dentition

**Fig. 8:**
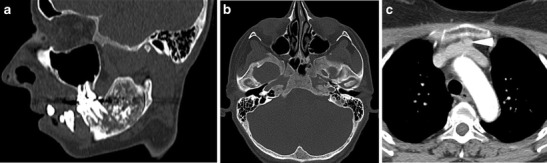
**a**) Sagittal oblique CT and **b**) axial CT image of the skull base **c**) axial CT upper thorax. 34-yr-old female with undiagnosed HPT-JT who underwent a CT to assess a left mandibular ramus lesion (a) subsequently proven to be an ossifying fibroma. She was incidentally noted to have generalised bony sclerosis (b) suggestive of hyperparathyroid bone disease and further imaging (c) revealed a retrosternal parathyroid adenoma (arrowhead) anterior to the left brachiocephalic vein
  In HPT-JT, parathyroid adenomas develop in a younger population with a mean age of diagnosis at 33 years, and there is a propensity for them to reoccur following removal as well as developing new adenomas [2]. In contrast, sporadic parathyroid adenomas typically occur as solitary single episode tumours beyond 40 years of age, with a predilection for the female population [9]. HPT-JT individuals are at a higher risk of developing parathyroid carcinoma, a rare malignancy in the general population [10].
  Parathyroid adenomas and carcinomas appear as solitary solid soft tissue masses the majority of which are juxtathyroid and located immediately posterior or inferior to the thyroid gland, although they can be located as inferior as the mediastinum [11]. First line assessment to identify parathyroid gland enlargement varies between institutions, but is typically performed with a combination of ultrasound and nuclear medicine scintigraphy. On ultrasound, parathyroid adenomas and carcinomas are uniformly hypoechoic and often demonstrate increased vascularity with identification of a vascular pedicle (Fig. 9) [11]. Nuclear medicine ‘wash out’ scinitigraphy using Technetium 99 m Sestamibi tracer is the ‘gold standard’ for the detection of parathyroid adenoma [12]. The tracer is retained within parathyroid adenoma and carcinomas and demonstrates increased uptake on both early and late imaging (Fig. 10). In contrast, there is rapid wash out of tracer from the adjacent thyroid gland [13]. CT and MRI are employed when there is non-concordance between first line imaging investigations and in patients who have typically undergone previous surgical intervention to remove a parathyroid adenoma/carcinoma [11]. Selective venous PTH sampling can be used to localize a suspected occult parathyroid adenoma.

**Fig. 9:**
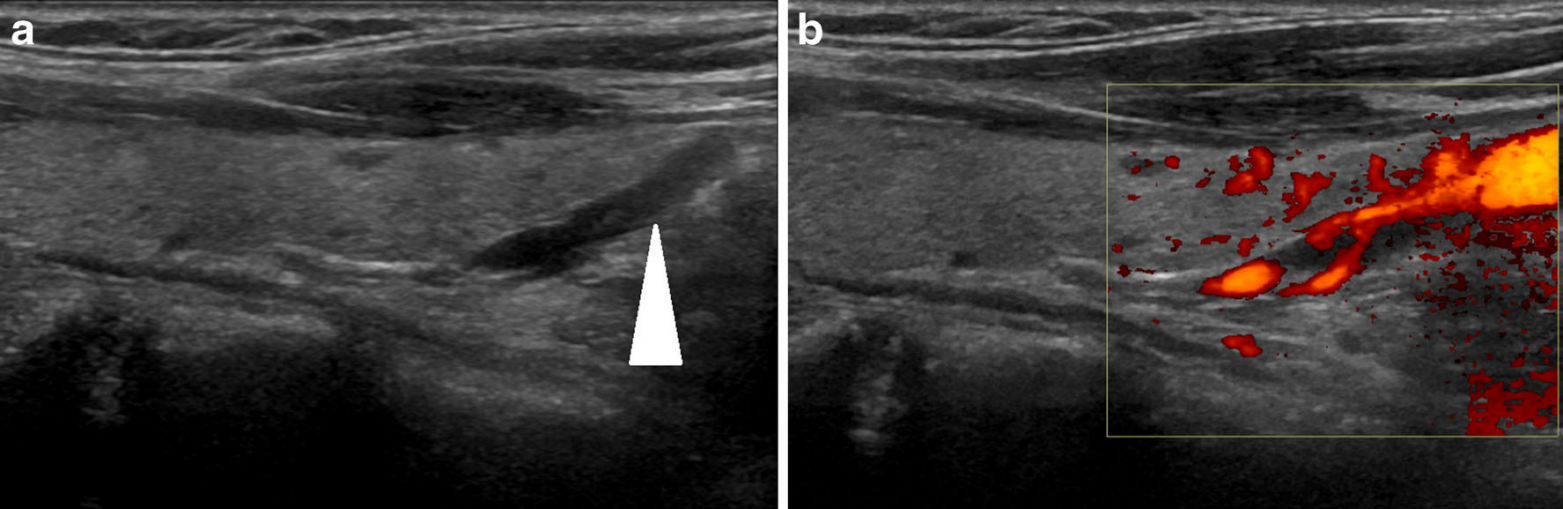
**a**) Longitudinal ultrasound image of a parathyroid adenoma in a 54-yr-old female with HPT-JT. The adenoma (arrowhead) corresponds to the elongated hypoechoic structure caudal to the thyroid. On application of colour Doppler (**b**) the lesion is demonstrated to be hypervascular

**Fig. 10:**
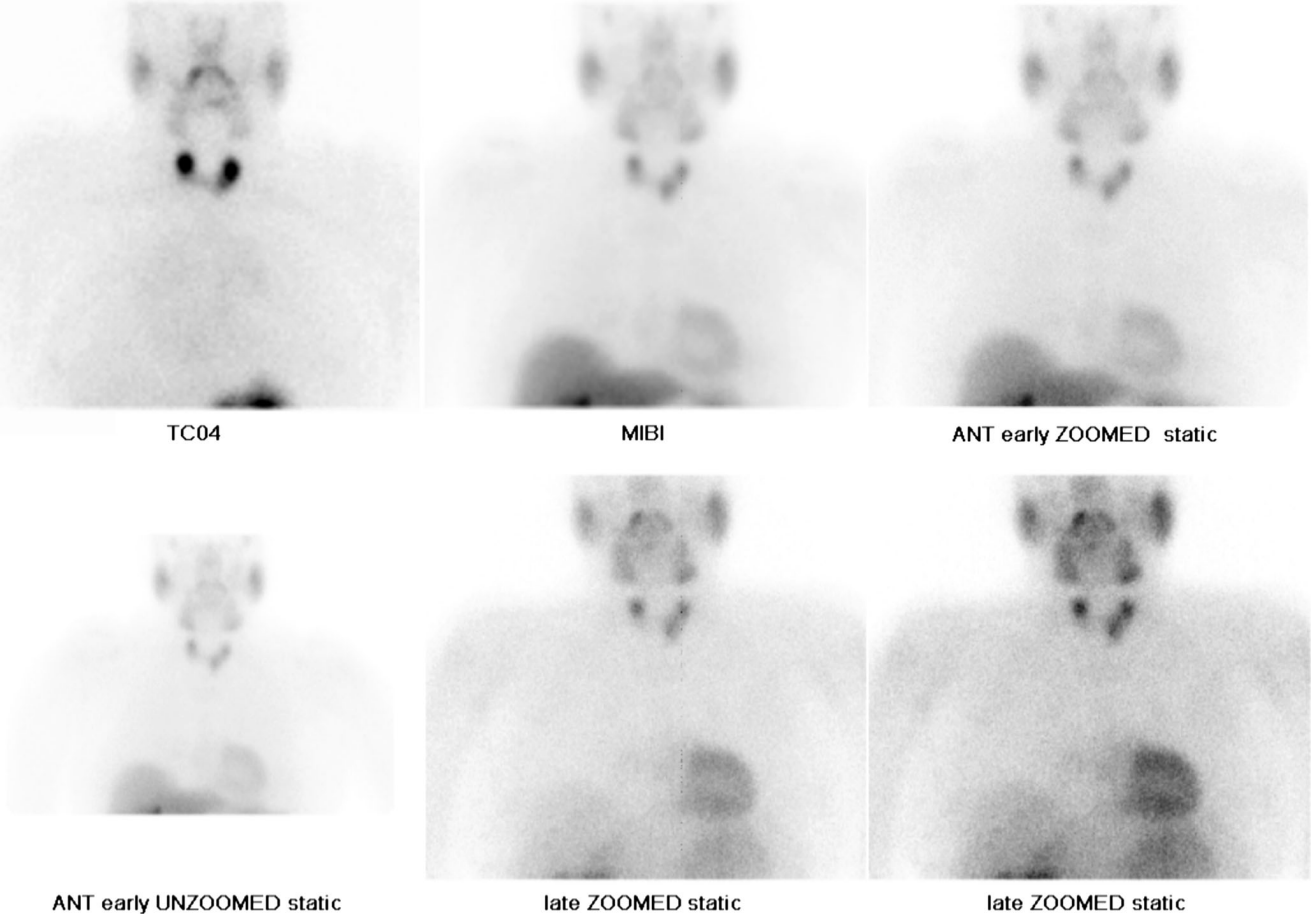
A nuclear medicine parathyroid scintigraphy study (image courtesy of Dr Cindy Leung, Barts and The London). The Technetium pertechnetate (^99m^Tc-pertechnetate) image (upper row, far left) demonstrates localization of the thyroid gland. ^99m^Tc-Sestamibi (MIBI) images show a focal area of significant increased tracer uptake inferior to the left thyroid lobe, which persists on early (centre and far right, upper row) and delayed imaging (centre and far right, lower row). Imaging characteristics are consistent with a parathyroid adenoma. Tracer uptake elsewhere is physiological
  Hyperparathyroidism and hypercalcaemia are often more profound in parathyroid carcinoma compared to parathyroid adenomas; however, the imaging appearances can be indistinguishable although parathyroid carcinomas are typically larger and may demonstrate invasive features, e.g., invasion of the prevertebral muscles and surrounding structures (Fig. 11) [14]. Therefore, in the absence of obvious metastatic disease, the diagnosis is typically confirmed following surgical resection.

**Fig. 11:**
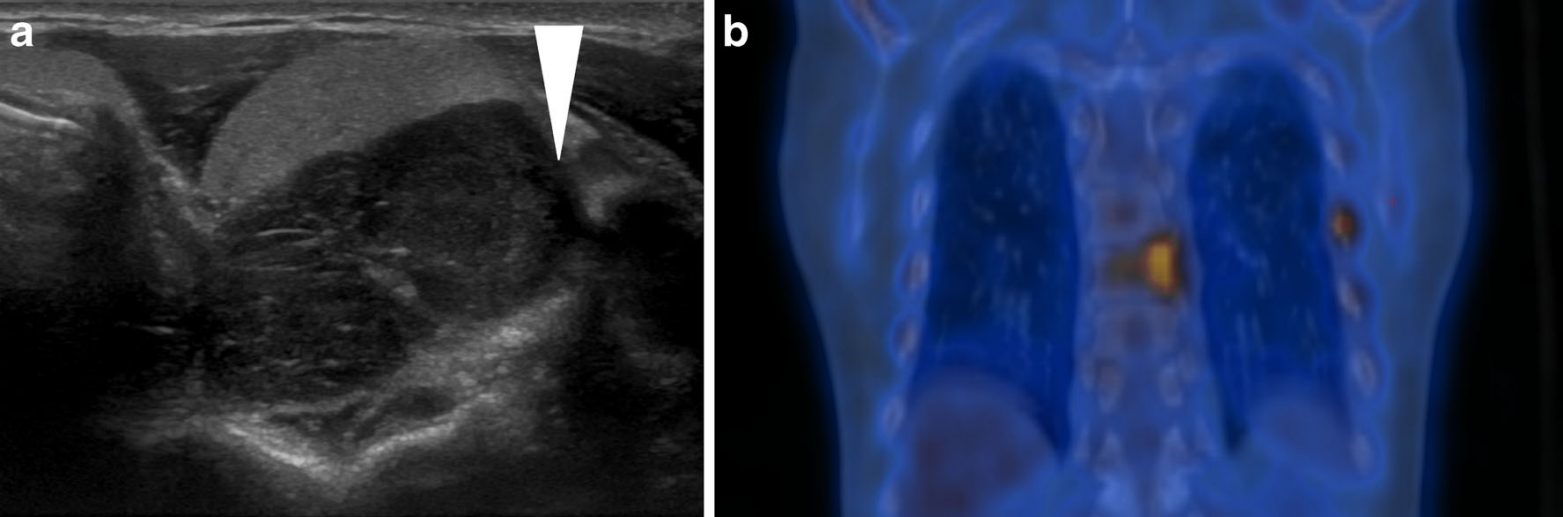
**a**) Transverse ultrasound image of thyroid and **b**) coronal FDG PET-CT of the thorax of a 50-year-old male with HPT-JT who has a parathyroid carcinoma and a bone metastasis at presentation. The ultrasound (a) demonstrates a hypoechoic parathyroid neoplasm (arrowhead) indenting the thyroid with findings suspicious for underlying invasion of the prevertebral musculature. The PET-CT demonstrates intense tracer uptake in the left posterior T8 vertebral body, which was proven on biopsy to be a metastatic deposit. Focal uptake in the left 7th rib is secondary to a rib fracture. HPT-JT patients have a greater predisposition to develop parathyroid carcinomas than the general population
iii. Renal and uterine tumours
  Wilms tumours, nephroblastomas, harmatomas, papillary renal cell carcinomas and an increased incidence of simple renal cysts have been reported in HPT-JT patients [15–17]. There is a reported higher incidence of uterine carcinomas and uterine polyposis in females with HPT-JT [17].

### Management

The management of ossifying fibromas in HPT-JT comprises complete surgical excision of jaw lesions with bone grafting and reconstruction. Patients with parathyroid lesions on imaging will often undergo a subtotal parathyroidectomy or total parathyroidectomy with autotransplatation [10]. The decision to perform total parathyroidectomy is based on consideration of the hormone levels and severity of parathyroid bone disease. HPT-JT individuals should be managed with a medical surveillance plan [10]. Plain film or CT can be used to detect jaw lesions, serum calcium measurements to detect parathyroid lesions and a combination of ultrasound and MRI can be used to evaluate the kidneys and uterus. Currently, there is no standardized management plan. Finally, family members can be screened genetically for the condition.

## Conclusion

HPT-JT is a rare syndrome with a significant potential for the development of facial disfigurement, morbidity secondary to hyperparathyroidism and malignancy. This topic is important for radiologists because patients will often first present with ossifying fibromas of the jaw. A high index of suspicion is required for the underlying diagnosis to be recognised, enabling treatment of hyperparathyroidism, early detection of malignant disease and screening of family members. Recurrent or multiple jaw lesions or the combination of jaw lesions and hyperparathyroid bone disease on imaging should raise the possibility of HPT-JT.
